# Case Report: Metastatic small bowel adenocarcinoma with DNA mismatch repair deficiency in an organ transplant recipient treated with anti-PD-1 immunotherapy

**DOI:** 10.3389/fonc.2025.1579364

**Published:** 2025-06-12

**Authors:** Quan H. Phung, Alexander K. Tsai, Byoung U. Park, Robben Schat, Richard Spong, L. Jill Tsai, Amit A. Kulkarni, Emmanuel S. Antonarakis, Arjun Gupta

**Affiliations:** ^1^ University of Minnesota, Minneapolis, MN, United States; ^2^ Guardant Health, Palo Alto, CA, United States; ^3^ Masonic Cancer Center, Minneapolis, MN, United States

**Keywords:** immunotherapy, DNA mismatch repair, tumor mutational burden, allograft transplant, gastrointestinal cancer

## Abstract

We present a case of a 65-year-old woman with a history of kidney and pancreas transplants for type 1 diabetes mellitus who presented with small bowel obstruction and was found to have a poorly differentiated small bowel adenocarcinoma with multifocal osseous and nodal metastases. Plasma-based next generation circulating tumor deoxyribonucleic acid (DNA) sequencing revealed mismatch repair deficiency and an exceptionally high tumor mutational burden (TMB) of 1069 mutations/megabase (mut/Mb). Initial management consisted of cytotoxic chemotherapy (FOLFOX; 5-fluorouracil, leucovorin, and oxaliplatin) given the urgent need for a clinical response. Following multidisciplinary discussion and shared decision-making, nivolumab was added with cycle 3 of FOLFOX. Transplant-related immunosuppression was adjusted, and pancreas and kidney transplant function were monitored closely. Potential organ rejection was monitored using donor-derived cell-free DNA. Immune-related adverse events were not observed. After 5 cycles of treatment (3 cycles involving nivolumab), she achieved a complete clinical, molecular, and radiographic response. There was minimal evidence of allograft rejection without signs of dysfunction. Treatment was discontinued and subsequent surveillance imaging suggested durable remission for at least 9 months following treatment cessation. This case highlights the importance of genomic testing and targeting actionable molecular alterations in patients with rare cancers, as well as the role of multidisciplinary care.

## Introduction

Small bowel adenocarcinoma (SBA) is a relatively uncommon, but aggressive, malignancy with dramatically rising incidence (1). Patients with metastatic disease are initially managed with multiagent cytotoxic chemotherapy. Though actionable mutations are rare, targeted agents are preferred in the second-line setting when available (2, 3). Additionally, a subset of SBA patients are eligible for immunotherapies, including immune checkpoint inhibitors (ICI). Specifically, ICI can be applied to patients with SBA tumors harboring DNA mismatch repair deficiencies (MMRd), high microsatellite instability (MSI-H), and/or elevated TMB. Approximately 15% of SBA tumors are MMRd/MSI-H, while ~10% have high TMB defined as ≥ 10 mutations/megabase (mut/Mb) (3, 4). While ICIs are ineffective in unselected SBA patients, response rates of 40-50% have been observed in MMRd/MSI-H patients (5–7). Pembrolizumab, a programmed death-1 (PD-1) inhibitor, gained tumor-agnostic approval for patients with TMB ≥10 mut/Mb based on KEYNOTE-158, though the trial did not specifically include patients with SBA (8). Rare cancer patients with “ultrahigh” TMB, defined as TMB ≥100 mut/Mb, have been described and are somewhat enriched in endometrial, colorectal, and other malignancies characterized by genomic instability (9–11). These tumors are often MMRd/MSI-H and/or harbor mutations in polymerase-encoding genes, such as DNA polymerase ϵ (*POLE*) or δ1 (*POLD1*) (9–12). However, such ultramutated cases have not been described in SBA.

Application of immunotherapies, including ICI, can be complicated by patient comorbid conditions, including autoimmune disorders and/or solid organ transplantation. Approximately half of patients with pre-existing autoimmune disorders will experience disease recurrence and/or symptom progression upon initiation of cancer treatment with ICI (8, 13–16). Likewise, nearly half of patients with solid organ transplants experience allograft rejection following ICI treatment (17). However, recent advances have defined immunosuppressive regimens that lower the risk of complications while maintaining ICI efficacy (18).

Here, we present a unique case of a patient with kidney and pancreas transplants with newly diagnosed metastatic, poorly differentiated small bowel adenocarcinoma and an exceptionally high blood TMB (>1000 mut/Mb) who was successfully treated with anti-PD-1 immunotherapy.

## Case description

A 65-year-old woman with a history of hypertension, hyperlipidemia, type 1 diabetes, and kidney and pancreas transplants presented to the emergency department with abdominal pain and vomiting. Simultaneous kidney and pancreas transplantation was performed approximately 15 years prior to presentation due to progressive diabetic complications, including neuropathy, retinopathy, diabetic coma, and worsening nephropathy with impending need for dialysis. Her chronic immunosuppression regimen prior to the index hospitalization included tacrolimus (goal 5–8 micrograms/liter), mycophenolic acid (360 mg every 12 hours), and prednisone (5 mg daily). There was a strong family history of cancer, including colorectal cancer in her father (diagnosed in his 60s), gastric cancer in her mother (diagnosed in her 40s), hepatocellular carcinoma in her brother (diagnosed in his 60s), a maternal uncle with pancreatic cancer (diagnosed in his 60s), and a maternal aunt with breast cancer (diagnosed in her 40s).

**Figure 1:**
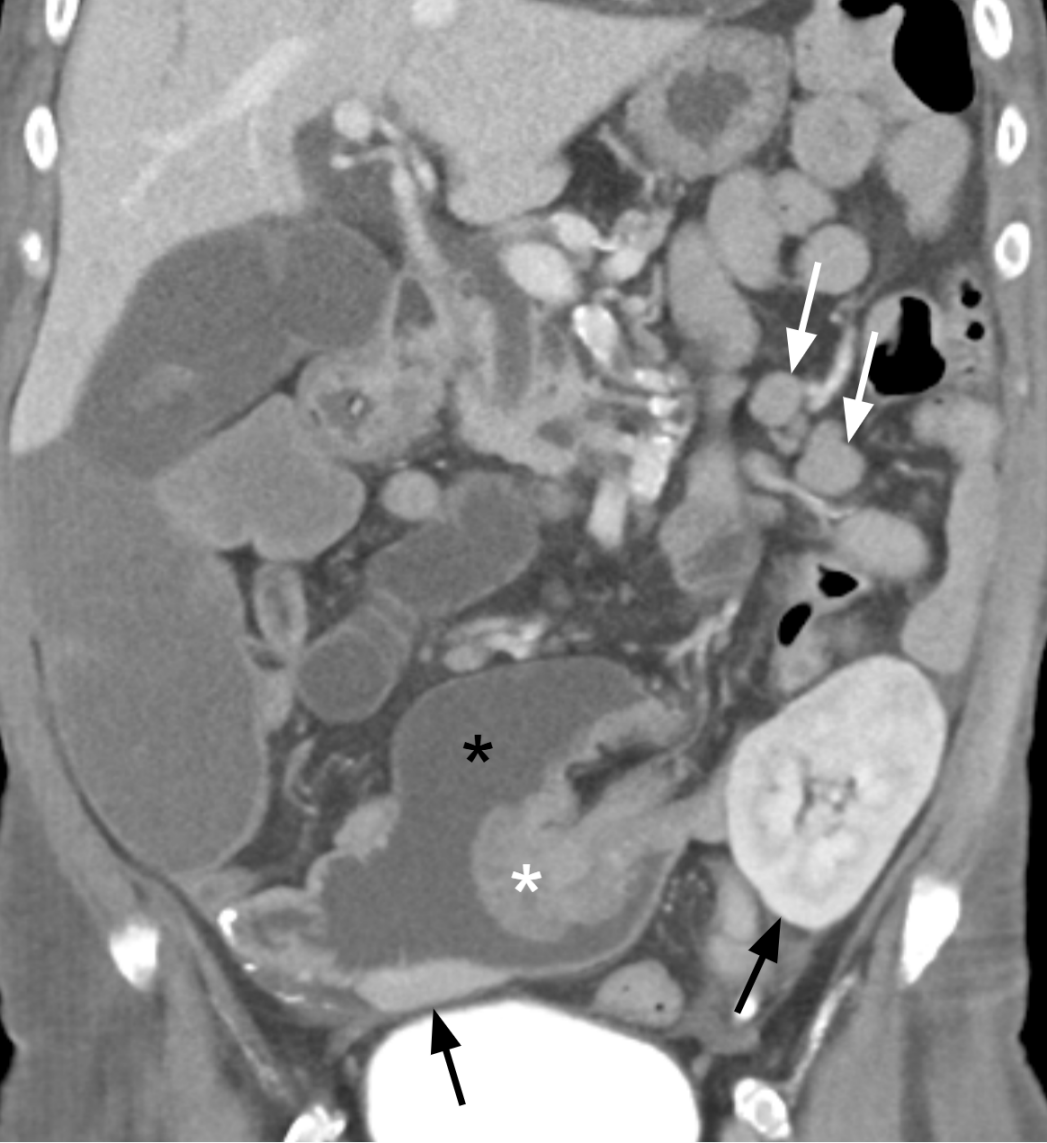
Coronal contrast enhanced portal venous phase CT image showing an enhancing lobulated intraluminal mass (white asterisk) measuring 4.6 x 4.0 x 4.1 cm arising from the small bowel wall acting as a lead point (intussusceptum) for an intussusception into the lumen of the right lower quadrant transplant pancreas duodenal cuff (i.e., intussuscipiens, black asterisk). Metastatic mesenteric lymphadenopathy is present in the left mid abdomen (white arrows). A small portion of the head of the transplant pancreas is observed in the right lower quadrant as well as a portion of the left lower quadrant renal transplant (black arrows), both of which demonstrate no radiographic abnormality.

**Figure 2:**
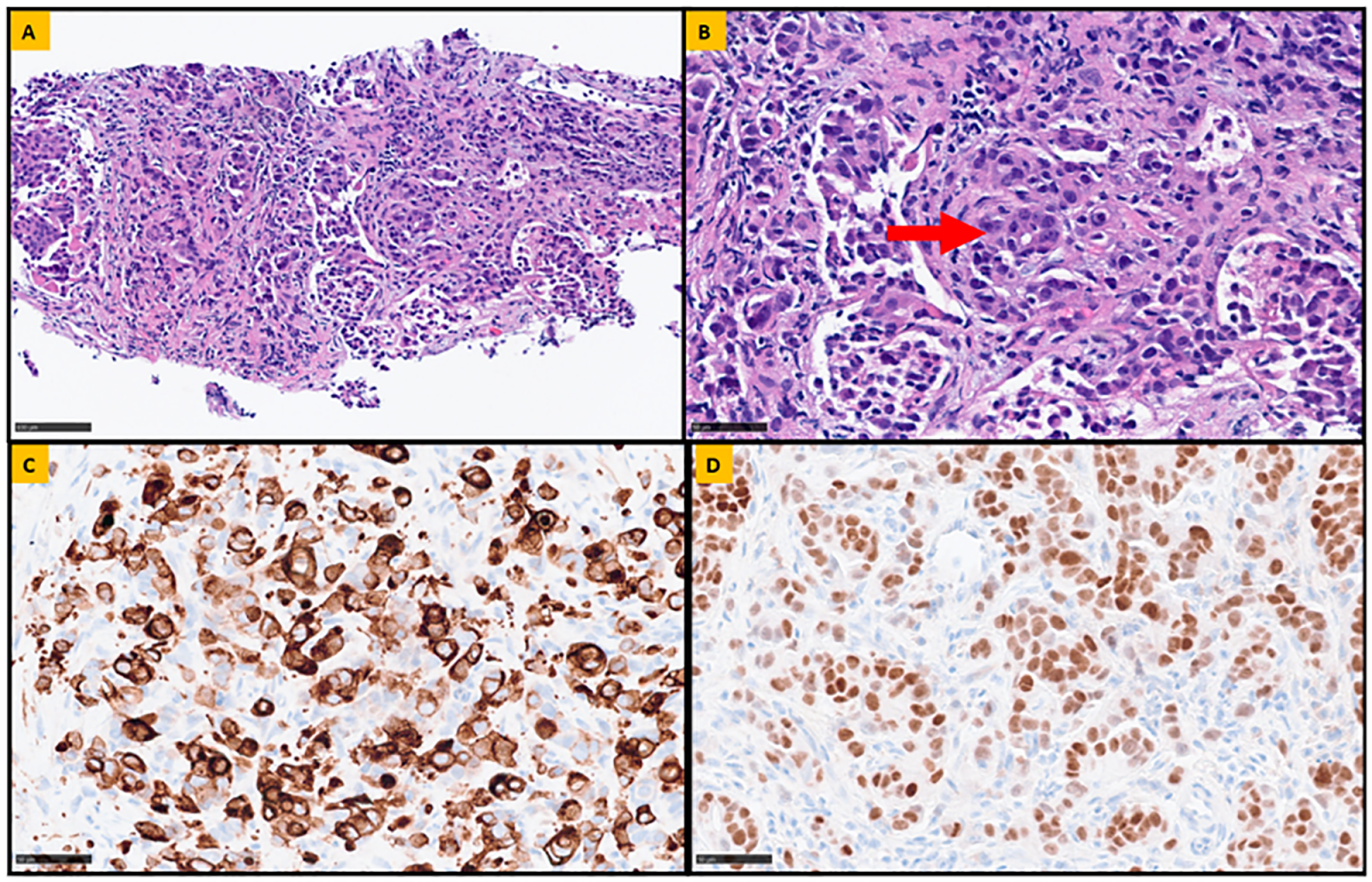
Representative sections of the retroperitoneal lymph node biopsy. **(A)** Fibroconnective tissue, infiltrated by atypical epithelial cells arranged in nests and single cells (Hematoxylin and Eosin stain, 20x magnification). **(B)** Rare focus of glandular differentiation (highlighted by the arrow) is also appreciated, confirming the diagnosis of metastatic poorly differentiated adenocarcinoma (Hematoxylin and Eosin stain, 40x magnification). To determine the potential site of origin, a panel immunohistochemical analysis was performed. The neoplastic cells were positive for Cytokeratin 7 (CK7; 40 x magnification) and Caudal-related Homeobox gene 2 (CDX-2; 40x magnification), shown in **(C, D)** respectively. Additionally, the lesional cells were negative for CK20, GATA-3, SATB2, PAX-8, SOX017, synaptophysin, chromogranin, HepPar-1, arginase, and HMB-45 (not shown).

A computed tomography (CT) scan of the abdomen and pelvis with intravenous contrast revealed a small bowel mass with associated intussusception and mesenteric lymphadenopathy (**Figure 1**), along with diffuse sclerotic osseous lesions and retroperitoneal lymphadenopathy. A retroperitoneal lymph node core biopsy revealed atypical epithelial cells arranged in nests and as single cells, with rare glandular differentiation, consistent with a poorly differentiated adenocarcinoma (**Figure 2**). Immunohistochemistry was positive for cytokeratin 7 (CK7) and Caudal-related Homeobox gene 2 (CDX-2), a profile most suggestive of an upper gastrointestinal primary neoplasm. Negative staining for a broad panel of other markers – including cytokeratin 20 (CK20), GATA-3, special AT-rich sequence-binding protein 2 (SATB2), paired-box gene 8 (PAX8), SRY-box transcription factor 17 (SOX17), synaptophysin, chromogranin, hepatocyte paraffin 1 (HepPar-1), arginase, and human melanoma black 45 (HMB-45), further excluded neoplasms from other common primary sites. Her diagnosis was most consistent with metastatic poorly differentiated small bowel adenocarcinoma. Evaluation of MMR proteins: MutL homolog 1 gene (MLH1), MutS homolog 2 (MSH2), MutS homolog 6 (MSH6), and postmeiotic segregation increased 2 (PMS2) by immunohistochemistry, as well as somatic molecular testing, including next generation sequencing (NGS) was unsuccessful due to insufficient amount of tissue. Thus, NGS was conducted via Guardent360^®^ plasma-based liquid biopsy (Guardant Health, Redwood City, CA). The assay reports single nucleotide variants, insertion and deletion variants (indels), fusions and copy number in up to 83 genes, as well as MSI status, and blood-based TMB (bTMB) (19–23). This test revealed pathogenic mutations in *MSH2* and *MSH6*, MSI-H status, and an exceptionally high TMB of 1069 mut/Mb, as well as a *POLE* E1977* variant detected with a mean allele fraction (MAF) of 0.67%. A full list of all pathogenic mutations detected by the Guardent360^®^ liquid-biopsy test is included in **Supplementary Table 1**. Subsequent germline testing was performed with Invitae Multi-Cancer Panel (Invitae, San Francisco, CA), which performs full-gene sequencing and deletion and duplication analysis using NGS to test 70 genes associated with cancers of varying organ systems. This did not reveal pathogenic inherited gene mutations, thus excluding Lynch syndrome.

Due to unremitting small bowel obstruction (SBO) despite nasogastric decompression and conservative measures, inpatient cytotoxic chemotherapy with FOLFOX was initiated to induce rapid cytoreduction. She received two doses of FOLFOX and subsequently developed evidence of anterograde bowel function resulting in hospital discharge. Extensive multidisciplinary discussions with the patient, medical oncology, and her transplant providers were completed, with conversations centered on the risks and benefits of adding immunotherapy to her regimen given her MMRd/MSI-H status and elevated TMB. The patient noted that she was not afraid of her cancer diagnosis or even mortality, but rather she was afraid of “not living life to the fullest.” For her, recurrent abdominal pain and complications from cancer significantly reduced her quality of life and kept her hospitalized and away from family. As there was a consensus for pursuing immunotherapy, intravenous nivolumab 240 mg every 2 weeks was added to coincide with FOLFOX treatments. Prior to receiving immunotherapy, donor-derived cell-free DNA (dd-cfDNA) was measured using the blood-based Prospera™ test (Natera, Austin, TX). This assay is used for solid organ transplant recipients and discriminates donor and patient DNA using single-nucleotide polymorphisms to report percentage of dd-cfDNA in the patient’s blood (24). The patient’s baseline dd-cfDNA prior to receiving immunotherapy was <0.08% [reference range: dd-cfDNA ≥ 1% associated with increased risk for transplant rejection]. This test would help serve as a reference point so that we could estimate how dd-cfDNA, and therefore the patient’s potential risk for allograph rejection, may change after receiving anti-PD1 immunotherapy.

**Figure 3:**
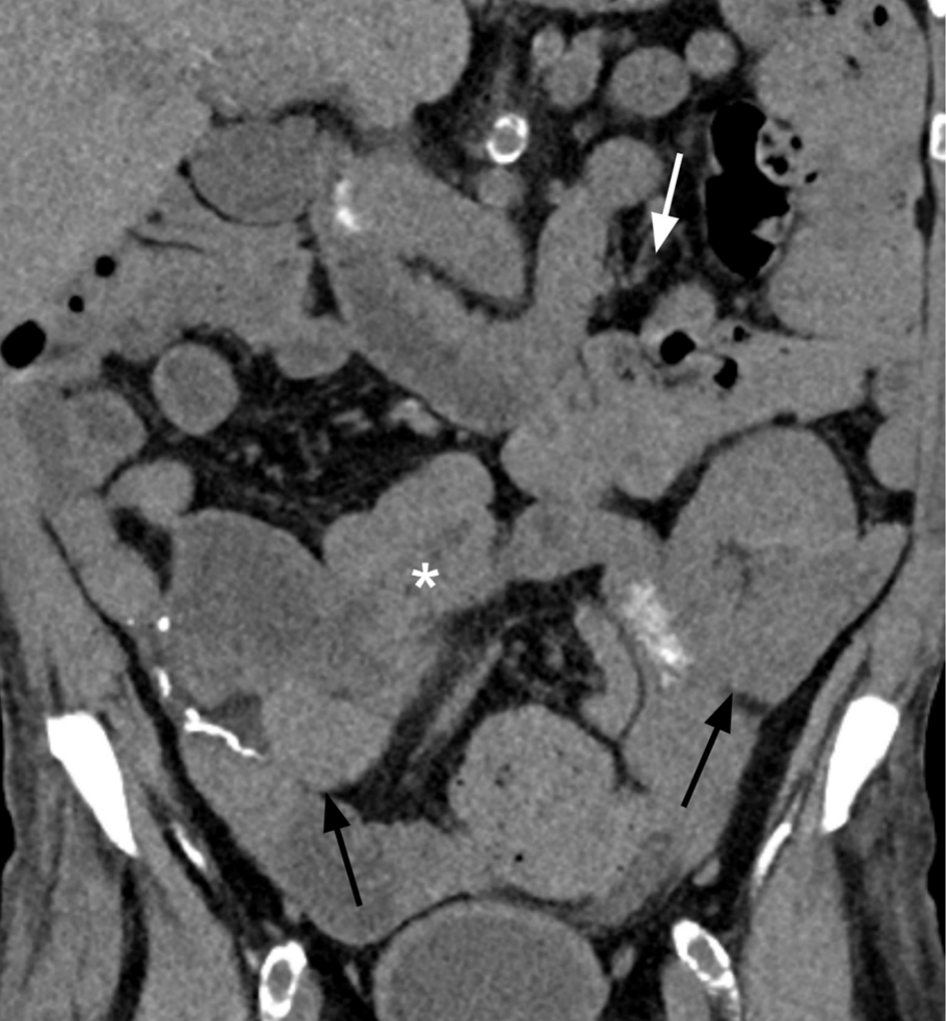
Follow-up coronal non-contrast CT image after 5 cycles of FOLFOX with 3 doses of nivolumab shows resolution of the small bowel mass and intussusception (white asterisk), resolution of the small bowel obstruction, and resolution of the mesenteric lymphadenopathy (white arrow). The renal and pancreas transplants demonstrated no new abnormality (black arrows).

**Figure 4:**
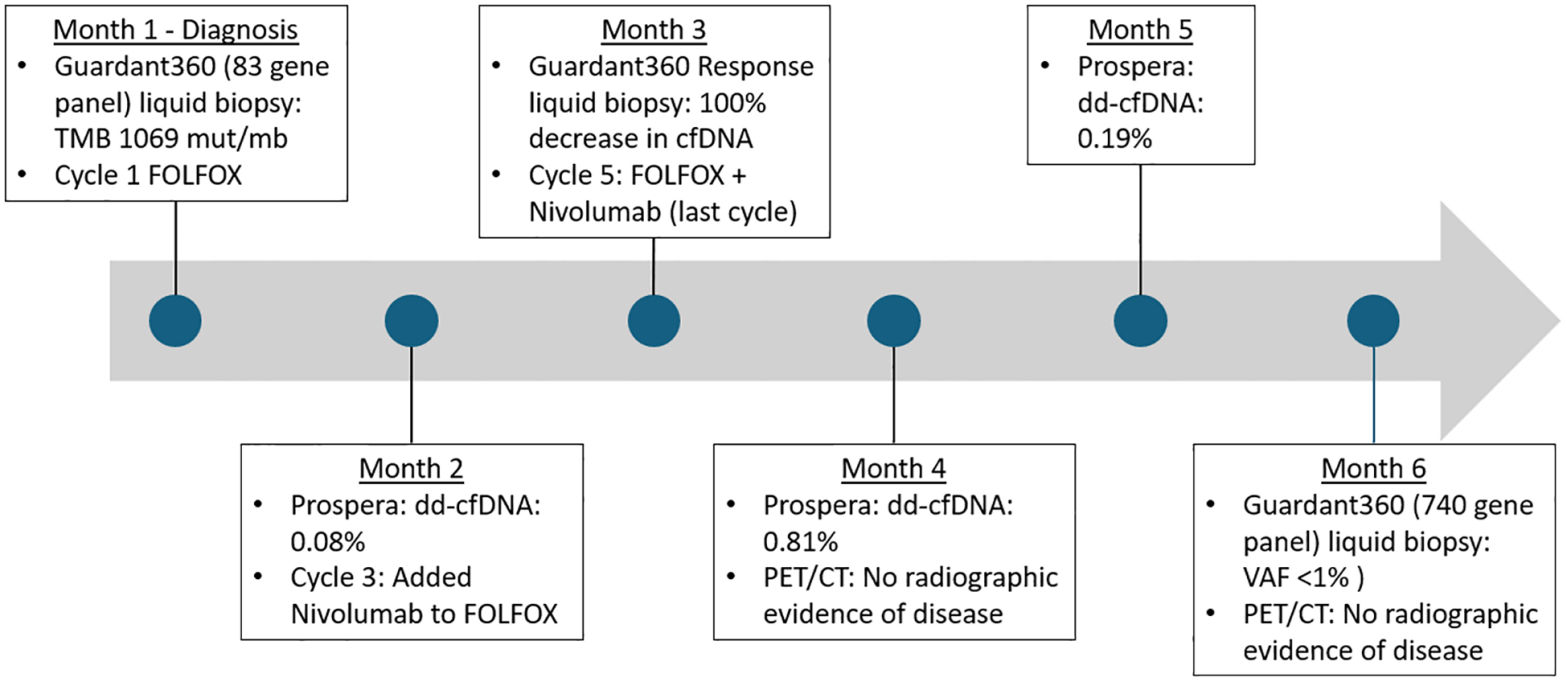
Timeline of treatment milestones and laboratory studies. Mut/Mb, mutations per megabase; dd-cfDNA, donor-derived cell-free DNA; cfDNA, cell-free DNA; VAF, variant allele fraction.

In anticipation of immunotherapy, her transplant team adjusted her immunosuppression regimen and tacrolimus and mycophenolic acid were replaced with everolimus (goal 4–6 micrograms/liter). Prednisone was also increased from 5 mg to 10 mg daily. After 2 cycles of FOLFOX, she received 3 cycles of chemoimmunotherapy with FOLFOX and nivolumab. A repeat liquid biopsy using Guardent360 Response^®^ (Guardant Health, Redwood City, CA) was performed after 4 cycles of treatment (including 2 cycles with nivolumab), which was approximately 2 months after starting systemic treatment. This assay is similar to Guardent360^®^ and also provides a molecular response score compared to a baseline test. This molecular response score is calculated as a ratio of mean variant allele frequencies between two timepoints, based on somatic single nucleotide variants, small insertion and deletion variants, and gene fusions (25). Testing revealed a 100% decrease in circulating tumor DNA (ctDNA), and the initial *MSH2* and *MSH6* mutations were undetectable. Furthermore, a repeat computer tomography (CT) scan of the chest, abdomen, and pelvis without intravenous contrast after a total of 5 cycles of treatment, showed resolution of her prior small bowel intussusception, decreased size and conspicuity of the associated small bowel mass, and resolved mesenteric and retroperitoneal lymphadenopathy (**Figure 3**). While increased sclerotic appearing osseous lesions were noted, a subsequent positron emission tomography (PET) with CT scan showed no evidence of metabolically active disease in the bone or other prior sites of disease, so these changes were felt to reflect treatment effect and bone healing. Together, these data were indicative of clinical, molecular, and radiographic complete response. An overview of the patient’s treatment, laboratory, and imaging milestones is provided in **Figure 4**.

Given the patient’s dramatic clinical and molecular response, multidisciplinary discussions with the patient then centered on the risks and benefits of further cancer-directed treatments. To aid in decision-making, Prospera™ testing was repeated after 5 cycles of treatment, which showed a dd-cfDNA of 0.81% [reference range: dd-cfDNA ≥ 1% associated with increased risk for transplant rejection]. Though remaining below the manufacturer’s 1% reference range for increased risk for rejection, dd-cfDNA was increased compared to the patient’s baseline of <0.08% prior to immunotherapy. Following repeat Prospera™ testing, her transplant team decided to increase immunosuppression by adding tacrolimus (goal 4–6 micrograms/liter), along with a reduction in prednisone to 5 mg daily, and continuing everolimus (goal 4–6 micrograms/liter). Following further discussions with the patient and her transplant team, in the context of evidence of subclinical allograft rejection and complete response, the decision was made to discontinue further cancer-directed therapies in favor of close surveillance.

Prospera™ dd-cfDNA testing was again performed three months after cessation of chemoimmunotherapy, which showed a decrease in dd-cfDNA to 0.19% suggestive of reduced risk of allograft rejection in the setting of chemoimmunotherapy cessation. Repeat PET/CT was completed four months after the final dose of chemoimmunotherapy and did not reveal evidence of recurrent disease. A repeat Guardant360^®^ (Guardant Health, Redwood City, CA) assay was performed four months after therapy complication. This is a 740-gene panel, which had been updated since her initial testing, that reports a methylation-based tumor fraction and identifies alterations in up to 740 genes associated with treatment decision-making. The Guardant360^®^ results showed no detection of *MSH2* and *MSH6* mutations and a negative MSI-H status. The somatic variants *GNAS* Q227H and *ESR1* R269C were both detected at a VAF below 1%. Guardant360^®^ also includes a novel classifier that combines genomics, methylation, and fragmentomics to distinguish variants of potential clonal hematopoiesis in plasma samples with >98% specificity. The variants *DNMT3A* W709* (VAF 0.3%), *GNAS* R201H (VAF 0.09%), *NF1* D2346G (VAF 0.2%), and *SMO* N309S (VAF 0.2%) were all reported as variants of potential clonal hematopoiesis. The patient continues to have no evidence of disease at the time of publication (9 months after cessation of systemic therapy). In the interim, the patient regained significant physical capacity, has resumed part-time employment, and is living a full life. Continued surveillance is anticipated, including repeat PET/CT and Guardant Health liquid biopsy every 3 months.

## Discussion

While the rarity of SBA has limited collective knowledge, recent studies have begun to characterize the molecular drivers of SBA tumorigenesis, some of which are clinically actionable (3, 4). These studies have also revealed that signatures of genomic instability, including MMRd/MSI-H and high TMB, are relatively common in SBA. Reported MMRd/MSI-H incidence rates are similar to those noted in colorectal cancer and gynecological cancers, where these deficiencies are most frequent (26, 27). These genomic instabilities are thought to increase expression of neoantigens, which can be detected by the immune system (28). Tissue or histology agnostic treatment options have become increasingly relevant, especially among gastrointestinal malignancies (29, 30). Notably, pembrolizumab first gained Food and Drug Administration (FDA) accelerated tumor-agnostic approval for MMRd/MSI-H malignancies in 2017 based on results from KEYNOTE-016 (31). Full FDA approval was subsequently granted in 2023. Dostarlimab, which also targets PD-1, gained initial FDA approval for MMRd/MSI-H patients in 2021 based on the GARNET trial (6). Both trials demonstrated approximately 40% objective response rates (ORR).

Pembrolizumab is also approved for patients with high TMB based on results from the KEYNOTE-158 trial. SBA was not represented in the trial, and ICI responses in SBA patients with high TMB have not been reported. However, a subset (~10%) of SBA patients appear to harbor tumors with high TMB (3). MMRd/MSI-H malignancies are associated with high TMB (9). Mutations in DNA repair pathways such as *BRCA1/2* also result in slight increases in TMB (32, 33). A representative case report of this finding describes a patient with a metastatic ampullary cancer with BRCA2 germline mutation and TMB of 11 mut/Mb, who actually had a marked response to chemotherapy (34). There is also early research that certain medications can influence MMRd or increased TMB expression (35). More striking elevations in TMB have been noted in patients with mutations in *POLE* and *POLD1*. These genes, which encode DNA polymerases, contain polymerase and exonuclease domains, the latter of which performs a proofreading function that is essential to maintain DNA fidelity during DNA replication. Loss of function mutations, which usually occur in the exonuclease domain, abrogate this proofreading function. The resultant accumulation of mutations can result in markedly elevated TMB >100 mut/Mb, often referred to as ultrahigh TMB (9–11). Elevated levels of TMB in this range are rare in cancer – even among MMRd bowel cancers there are typically less than 5% with a TMB >100 mut/Mb and less than 1% with TMB >500 mut/Mb (9, 36).

To our knowledge, the case detailed herein is the first reported case with a TMB greater than 100 mut/Mb in SBA. The patient’s SBA harbored an exceptional TMB of 1,069 mut/Mb in the setting of both MMRd/MSI-H disease and a variant in *POLE*. Notably, while MMRd/MSI-H status alone is associated with high TMB, levels higher than 100 mut/Mb are rare (6). This raises the possibility that the patient’s remarkable mutational burden resulted from a combination of MMRd/MSI-H and loss of function in *POLE*, as has been reported previously (9, 10, 37). However, the *POLE* E1977* variant has only been reported in a single additional instance and was associated with a TMB of 182 mut/Mb, though its rarity precluded classification as a pathogenic variant (9). Indeed, large cohorts are required to validate rare *POLE* variants as pathogenic. Further, this variant does not lie within the proofreading (exonuclease) domain that helps maintain genome integrity and where most pathogenic mutations occur, and in this patient’s case was identified with a low MAF of 0.67% (10). Thus, the mechanisms underlying development of such profoundly elevated mutational burden remain unclear. Notably, though blood-based TMB (bTMB) measurements performed on ctDNA are positively correlated with tissue-derived TMB (tTMB), concordance is limited and some studies estimate that bTMB can be 2–3 times higher than tTMB (38–41). Though the patient therefore could not be classified as ultrahigh TMB, the elevated TMB and dramatic response to an ICI are in line with other reports of immunotherapy for patients with high TMB (12).

Patients with organ transplants are a vulnerable population who must balance appropriate levels of immunosuppression and risk for infection and other complications. Treatment with ICI carries significant risk for allograft rejection. One retrospective study reported allograft rejection in 41% of patients after receiving an ICI (17). Relatedly, a systematic review encompassing reports between 2014 and 2017 reported evidence against the use of ICI due to the high risk of allograft rejection, although this included a relatively small number of patients (20 cases with 12 allograft rejections) (42).

The intersectionality between oncology and solid organ transplantation can be difficult to navigate as the goals for immunosuppression required for organ retention and immune stimulation required for anticancer efficacy conflict with one another. One literature review showed that in patients with solid organ transplants who received immunotherapy, less than one third (30.8%) of patients achieved the preferred outcome of effective immunotherapy with retained transplant (43). As such, oncologists are often hesitant to use ICI in patients with solid organ transplants, preferring to save immunotherapy for situations without suitable alternative treatment options. For this patient, we were initially hesitant to add an ICI to her systemic therapy regimen, preferentially treating with chemotherapy. However, given recurrent small bowel obstructions resulting in prolonged hospitalization and poor quality of life, our decision calculus and the patient’s preference shifted in favor of incorporating immunotherapy. This decision was also influenced by the detection of MMRd/MSI-H status and high TMB.

Once the decision is made to treat a patient with a solid organ transplant with immunotherapy, the question arises about how to detect allograft rejection. An area of research being pioneered in fields such as oncology and obstetrics involves evaluating cfDNA, which is fragmented extracellular DNA that is released into the bloodstream from cells undergoing apoptosis. In transplantation medicine, dd-cfDNA uses this concept to measure fragmented DNA from the donor allograft as an early indicator for organ rejection. Recent studies have shown growing evidence that increased dd-cfDNA is associated with organ rejection, with increased levels of dd-cfDNA being predictive of decline in estimated glomerular filtration rate (eGFR), and increase risk for developing donor specific antibodies or T-cell mediated rejection (44, 45). In practice, there is not yet a gold standard dd-cfDNA level that should provoke a clinical change in management. Early studies indicate that a dd-cfDNA level <1% suggests an absence of active rejection, while a level >1% indicates a higher probability of active rejection (46, 47). In the presented case, dd-cfDNA was measured prior to immunotherapy initiation as well as two months later, after receiving three cycles of chemotherapy with nivolumab. On both occasions, the dd-cfDNA was below the reference target of <1%, however there was a noticeable rise in her dd-cfDNA from <0.08 to 0.81 percent, which led to alterations in the patient’s immunosuppressive regimen.

Since we would expect that the risk for allograft rejection would increase with prolonged ICI treatment, we utilized molecular and radiographic approaches to identify residual disease following initial ICI treatment. A repeat liquid biopsy (Guardant360^®^ assay, assessing up to 740 genes) after the patient’s fourth cycle of treatment showed continued decline of ctDNA and undetectable *MSH2* and *MSH6* mutations. Studies have suggested that ctDNA can help characterize the molecular profile of a tumor and be utilized to screen for early recurrence (48, 49). Additionally, longitudinal testing may help screen for clonal changes and risk for treatment resistance (50). Relatedly, molecular response assessment based on ctDNA can predict improved progression-free survival and overall survival compared to patients without a molecular response (51, 52). Notably, molecular studies correlated with multiple imaging evaluations, and concordant evidence for this patient was suggestive of a complete response. Furthermore, this response was achieved rapidly, within 2–3 months of starting immunotherapy. Thus, in the context of potential further risks to allograft maintenance, we ultimately opted to stop cancer-directed therapies in favor of close surveillance. There is limited data and no formal protocol defining optimal management of patients in this situation. Surveillance will be completed with PET/CT and Guardant Health liquid biopsy NGS testing every 3 months. Thus far, more than 11 months after her initial diagnosis, she continues to be in complete clinical and molecular remission.

Findings from our single patient case report are difficult to generalize more broadly to SBA patients with high TMB. Additionally, there are limited formal quantitative analyses as part of this study. While many of the concepts discussed and rationale for clinical changes may be informative, the specifics may not necessarily be applicable to other patients. Although this patient experienced a complete response, a positive outcome was not guaranteed and a separate patient may have faced significant adverse effects from anti-PD-1 immunotherapy and/or lack of immunotherapy efficacy. Moreover, we were unable to perform DNA mutational signature analyses which could define the relative contributions of MMRd/MSI-H and mutated *POLE* to the observed elevated TMB (53). Nevertheless, this case highlights how molecular testing has the potential to expand unique treatment options for patients in difficult clinical situations, particularly when malignant tissue is unavailable or insufficient. Future studies will focus on characterizing the impact of rare *POLE* and *POLD1* mutations, such as the described *POLE* E1977* variant, to somatic hypermutation in cancer. Interestingly, many of the *POLE* and *POLD1* mutations that appear to contribute to mutation accumulation do not encode DNA within the exonuclease domains (54). Further research is also needed to develop guidelines for use of immunotherapy in patients with allografts and to accurately assess risk for transplant rejection.

## Conclusion

We present a case of a patient with aggressive, metastatic, poorly differentiated small bowel adenocarcinoma who had recurrent episodes of small bowel obstruction. She was found to have MMR deficiency, microsatellite instability, and high TMB (>1000 mut/Mb) as reported from liquid biopsy. Thus, in addition to standard-of-care chemotherapy (FOLFOX), she was a unique candidate for anti-PD-1 immunotherapy. However, her history of kidney and pancreas transplants made this a perilous proposition due to concerns for allograph rejection. Despite initiating chemotherapy, she had a prolonged hospitalization with recurrent episodes of abdominal pain due to small bowel obstruction, which prompted the addition of nivolumab at cycle 3. Although collective knowledge on dd-cfDNA is still evolving, it was used here to inform on potential risk for allograft rejection in this unique scenario. She had an excellent response after 5 cycles of systemic therapy (2 cycles with FOLFOX, 3 cycles with FOLFOX plus nivolumab) and achieved a complete remission with this chemoimmunotherapy regimen. While there are certainly risks involved with using immunotherapy in the organ-transplant setting, the reward can be especially advantageous for individuals with MSI-H status and/or high TMB.

## Data Availability

The original contributions presented in the study are included in the article/**Supplementary Material**. Further inquiries can be directed to the corresponding author.

## References

[1] Khosla D Dey T Madan R Gupta R Goyal S Kumar N. Small bowel adenocarcinoma: An overview. World J Gastrointest Oncol. (2022) 14:413–22. doi: 10.4251/wjgo.v14.i2.413 PMC8918997 35317322

[2] Schrock AB Devoe CE McWilliams R Sun J Aparicio T Stephens PJ. Genomic profiling of small-bowel adenocarcinoma. JAMA Oncol. (2017) 3:1546–53. doi: 10.1001/jamaoncol.2017.1051 PMC5710195 28617917

[3] Gelsomino F Balsano R De Lorenzo S Garajová I. Small bowel adenocarcinoma: from molecular insights to clinical management. Curr Oncol. (2022) 29:1223–36. doi: 10.3390/curroncol29020104 PMC8870676 35200603

[4] Hänninen UA Katainen R Tanskanen T Plaketti RM Laine R Hamberg J. Exome-wide somatic mutation characterization of small bowel adenocarcinoma. PloS Genet. (2018) 14:e1007200. doi: 10.1371/journal.pgen.1007200 29522538 PMC5871010

[5] Pedersen KS Foster NR Overman MJ Boland PM Kim SS Arrambide KA. ZEBRA: A multicenter phase II study of pembrolizumab in patients with advanced small-bowel adenocarcinoma. Clin Cancer Res. (2021) 27:3641–8. doi: 10.1158/1078-0432.CCR-21-0159 33883178

[6] André T Berton D Curigliano G Sabatier R Tinker AV Oaknin A. Antitumor activity and safety of dostarlimab monotherapy in patients with mismatch repair deficient solid tumors: A nonrandomized controlled trial. JAMA Netw Open. (2023) 6:e2341165. doi: 10.1001/jamanetworkopen.2023.41165 37917058 PMC10623195

[7] Marabelle A Le DT Ascierto PA Di Giacomo AM De Jesus-Acosta A Delord JP. Efficacy of pembrolizumab in patients with noncolorectal high microsatellite instability/mismatch repair-deficient cancer: results from the phase II KEYNOTE-158 study. J Clin Oncol. (2020) 38:1–10. doi: 10.1200/JCO.19.02105 31682550 PMC8184060

[8] Marabelle A Fakih M Lopez J Shah M Shapira-Frommer R Nakagawa K. Association of tumour mutational burden with outcomes in patients with advanced solid tumours treated with pembrolizumab: prospective biomarker analysis of the multicohort, open-label, phase 2 KEYNOTE-158 study. Lancet Oncol. (2020) 21:1353–65. doi: 10.1016/S1470-2045(20)30445-9 32919526

[9] Campbell BB Light N Fabrizio D Zatzman M Fuligni F de Borja R. Comprehensive analysis of hypermutation in human cancer. Cell. (2017) 171:1042–56.e10. doi: 10.1016/j.cell.2017.09.048 29056344 PMC5849393

[10] Haradhvala NJ Kim J Maruvka YE Polak P Rosebrock D Livitz D. Distinct mutational signatures characterize concurrent loss of polymerase proofreading and mismatch repair. Nat Commun. (2018) 9:1746. doi: 10.1038/s41467-018-04002-4 29717118 PMC5931517

[11] He J Ouyang W Zhao W Shao L Li B Liu B. Distinctive genomic characteristics in POLE/POLD1-mutant cancers can potentially predict beneficial clinical outcomes in patients who receive immune checkpoint inhibitor. Ann Transl Med. (2021) 9:129. doi: 10.21037/atm-20-7553 33569431 PMC7867935

[12] Garmezy B Gheeya J Lin HY Huang Y Kim T Jiang X. Clinical and molecular characterization of POLE mutations as predictive biomarkers of response to immune checkpoint inhibitors in advanced cancers. JCO Precis Oncol. (2022) 6:e2100267. doi: 10.1200/PO.21.00267 35108036 PMC8820927

[13] Xie W Huang H Xiao S Fan Y Deng X Zhang Z. Immune checkpoint inhibitors therapies in patients with cancer and preexisting autoimmune diseases: A meta-analysis of observational studies. Autoimmun Rev. (2020) 19:102687. doi: 10.1016/j.autrev.2020.102687 33131688

[14] Tison A Garaud S Chiche L Cornec D Kostine M. Immune-checkpoint inhibitor use in patients with cancer and pre-existing autoimmune diseases. Nat Rev Rheumatol. (2022) 18:641–56. doi: 10.1038/s41584-022-00841-0 36198831

[15] Sparks JA. Pre-existing autoimmune diseases and immune checkpoint inhibitors for cancer treatment: considerations about initiation, flares, immune-related adverse events, and cancer progression. Rheum Dis Clin North Am. (2024) 50:147–59. doi: 10.1016/j.rdc.2024.01.001 38670718

[16] Ibis B Aliazis K Cao C Yenyuwadee S Boussiotis VA. Immune-related adverse effects of checkpoint immunotherapy and implications for the treatment of patients with cancer and autoimmune diseases. Front Immunol. (2023) 14:1197364. doi: 10.3389/fimmu.2023.1197364 37342323 PMC10277501

[17] Abdel-Wahab N Safa H Abudayyeh A Johnson DH Trinh VA Zobniw CM. Checkpoint inhibitor therapy for cancer in solid organ transplantation recipients: an institutional experience and a systematic review of the literature. J Immunother Cancer. (2019) 7:106. doi: 10.1186/s40425-019-0585-1 30992053 PMC6469201

[18] Murakami N Mulvaney P Danesh M Abudayyeh A Diab A Abdel-Wahab N. A multi-center study on safety and efficacy of immune checkpoint inhibitors in cancer patients with kidney transplant. Kidney Int. (2021) 100:196–205. doi: 10.1016/j.kint.2020.12.015 33359528 PMC8222056

[19] Lanman RB Mortimer SA Zill OA Sebisanovic D Lopez R Blau S. Analytical and clinical validation of a digital sequencing panel for quantitative, highly accurate evaluation of cell-free circulating tumor DNA. PloS One. (2015) 10:e0140712. doi: 10.1371/journal.pone.0140712 26474073 PMC4608804

[20] Odegaard JI Vincent JJ Mortimer S Vowles JV Ulrich BC Banks KC. Validation of a plasma-based comprehensive cancer genotyping assay utilizing orthogonal tissue- and plasma-based methodologies. Clin Cancer Res. (2018) 24:3539–49. doi: 10.1158/1078-0432.CCR-17-3831 29691297

[21] Willis J Lefterova MI Artyomenko A Kasi PM Nakamura Y Mody K. Validation of microsatellite instability detection using a comprehensive plasma-based genotyping panel. Clin Cancer Res. (2019) 25:7035–45. doi: 10.1158/1078-0432.CCR-19-1324 31383735

[22] Rizvi NA Cho BC Reinmuth N Lee KH Luft A Ahn M-J. Durvalumab with or without tremelimumab vs standard chemotherapy in first-line treatment of metastatic non–small cell lung cancer: the MYSTIC phase 3 randomized clinical trial. JAMA Oncol. (2020) 6:661–74. doi: 10.1001/jamaoncol.2020.0237 PMC7146551 32271377

[23] Si H Kuziora M Quinn KJ Helman E Ye J Liu F. A blood-based assay for assessment of tumor mutational burden in first-line metastatic NSCLC treatment: results from the MYSTIC study. Clin Cancer Res. (2021) 27:1631–40. doi: 10.1158/1078-0432.CCR-20-3771 33355200

[24] Bromberg JS Bunnapradist S Samaniego-Picota M Anand S Stites E Gauthier P. Elevation of donor-derived cell-free DNA before biopsy-proven rejection in kidney transplant. Transplantation. (2024) 108:1994–2004. doi: 10.1097/TP.0000000000005007 38595232 PMC11335081

[25] Zhang Q Luo J Wu S Si H Gao C Xu W. Prognostic and predictive impact of circulating tumor DNA in patients with advanced cancers treated with immune checkpoint blockade. Cancer Discov. (2020) 10:1842–53. doi: 10.1158/2159-8290.CD-20-0047 PMC8358981 32816849

[26] Gutierrez C Ogino S Meyerhardt JA Iorgulescu JB. The prevalence and prognosis of microsatellite instability-high/mismatch repair-deficient colorectal adenocarcinomas in the United States. JCO Precis Oncol. (2023) 7:e2200179. doi: 10.1200/PO.22.00179 36716414 PMC9928756

[27] Zhao P Li L Jiang X Li Q. Mismatch repair deficiency/microsatellite instability-high as a predictor for anti-PD-1/PD-L1 immunotherapy efficacy. J Hematol Oncol. (2019) 12:54. doi: 10.1186/s13045-019-0738-1 31151482 PMC6544911

[28] Kelly RJ Bever K Chao J Ciombor KK Eng C Fakih M. Society for Immunotherapy of Cancer (SITC) clinical practice guideline on immunotherapy for the treatment of gastrointestinal cancer. J Immunother Cancer. (2023) 11:e006658. doi: 10.1136/jitc-2022-006658 37286304 PMC10254964

[29] Bhamidipati D Subbiah V. Impact of tissue-agnostic approvals for patients with gastrointestinal Malignancies. Trends Cancer. (2023) 9:237–49. doi: 10.1016/j.trecan.2022.11.003 PMC9974757 36494311

[30] Sartore-Bianchi A Agostara AG Patelli G Mauri G Pizzutilo EG Siena S. Application of histology-agnostic treatments in metastatic colorectal cancer. Dig Liver Dis. (2022) 54:1291–303. doi: 10.1016/j.dld.2022.05.013 35701319

[31] Le DT Uram JN Wang H Bartlett BR Kemberling H Eyring AD. PD-1 blockade in tumors with mismatch-repair deficiency. N Engl J Med. (2015) 372:2509–20. doi: 10.1056/NEJMoa1500596 PMC4481136 26028255

[32] Kensler KH Baichoo S Pathania S Rebbeck TR. The tumor mutational landscape of BRCA2-deficient primary and metastatic prostate cancer. NPJ Precis Oncol. (2022) 6:39. doi: 10.1038/s41698-022-00284-6 35715489 PMC9205939

[33] Seeber A Zimmer K Kocher F Puccini A Xiu J Nabhan C. Molecular characteristics of BRCA1/2 and PALB2 mutations in pancreatic ductal adenocarcinoma. ESMO Open. (2020) 5:e000942. doi: 10.1136/esmoopen-2020-000942 33229504 PMC7684832

[34] Mauri G Gori V Patelli G Roazzi L Rizzetto F De Carlis L. Multimodal treatment with curative intent in a germline BRCA2 mutant metastatic ampullary adenocarcinoma: a case report. World J Surg Oncol. (2023) 21:118. doi: 10.1186/s12957-023-02976-0 36998040 PMC10064505

[35] Crisafulli G Sartore-Bianchi A Lazzari L Pietrantonio F Amatu A Macagno M. Temozolomide treatment alters mismatch repair and boosts mutational burden in tumor and blood of colorectal cancer patients. Cancer Discov. (2022) 12:1656–75. doi: 10.1158/2159-8290.CD-21-1434 PMC9394384 35522273

[36] Ali-Fehmi R Krause HB Morris RT Wallbillich JJ Corey L Bandyopadhyay S. Analysis of concordance between next-generation sequencing assessment of microsatellite instability and immunohistochemistry-mismatch repair from solid tumors. JCO Precis Oncol. (2024) 8:e2300648. doi: 10.1200/PO.23.00648 39565978 PMC11594015

[37] Shah SM Demidova EV Ringenbach S Faezov B Andrake M Gandhi A. Exploring co-occurring POLE exonuclease and non-exonuclease domain mutations and their impact on tumor mutagenicity. Cancer Res Commun. (2024) 4:213–25. doi: 10.1158/2767-9764.CRC-23-0312 PMC10812383 38282550

[38] Sturgill E Misch A Jones C Luckett D Fu X Jones S. Concordance of blood and tissue TMB from NGS testing in real-world settings and their ability to predict response to immunotherapy. J Clin Oncol. (2021) 39:2540–. doi: 10.1200/JCO.2021.39.15_suppl.2540

[39] Franses JW Lim M Burgoyne AM Mody K Lennerz J Chang J. Profile and predictors of blood tumor mutational burden in advanced hepatocellular carcinoma. Oncologist. (2022) 27:e908–e11. doi: 10.1093/oncolo/oyac189 PMC9632309 36103364

[40] Mishima S Nakamura Y Tukachinsky H Taniguchi H Kadowaki S Kato K. Validity and utility of blood tumor mutational burden (bTMB) is dependent on circulating tumor DNA (ctDNA) shed: SCRUM-Japan MONSTAR-SCREEN. J Liquid Biopsy. (2023) 1:100003. doi: 10.1016/j.jlb.2023.100003 PMC11863975 40027285

[41] Park J Park I Hwang JY Bae W Lee G Kim L. Real-world concordance between tumor mutational burden from blood and tissue in lung cancer and other cancers. J Thorac Oncol. (2021) 16:S1023–S4. doi: 10.1016/j.jtho.2021.08.354

[42] Aguirre LE Guzman ME Lopes G Hurley J. Immune checkpoint inhibitors and the risk of allograft rejection: A comprehensive analysis on an emerging issue. Oncologist. (2019) 24:394–401. doi: 10.1634/theoncologist.2018-0195 30413665 PMC6519766

[43] Rünger A SChadendorf D Hauschild A Gebhardt C. Immune checkpoint blockade for organ-transplant recipients with cancer: A review. Eur J Cancer. (2022) 175:326–35. doi: 10.1016/j.ejca.2022.08.010 36191571

[44] Bu L Gupta G Pai A Anand S Stites E Moinuddin I. Clinical outcomes from the Assessing Donor-derived cell-free DNA Monitoring Insights of kidney Allografts with Longitudinal surveillance (ADMIRAL) study. Kidney Int. (2022) 101:793–803. doi: 10.1016/j.kint.2021.11.034 34953773

[45] Aubert O Ursule-Dufait C Brousse R Gueguen J Racapé M Raynaud M. Cell-free DNA for the detection of kidney allograft rejection. Nat Med. (2024) 30:2320–7. doi: 10.1038/s41591-024-03087-3 PMC11333280 38824959

[46] Bloom RD Bromberg JS Poggio ED Bunnapradist S Langone AJ Sood P. Cell-free DNA and active rejection in kidney allografts. J Am Soc Nephrol. (2017) 28:2221–32. doi: 10.1681/ASN.2016091034 PMC5491290 28280140

[47] Lakhani L Alasfar S Bhalla A Aala A Rosenberg A Ostrander D. Utility of serial donor-derived cell-free DNA measurements for detecting allograft rejection in a kidney transplant recipient after PD-1 checkpoint inhibitor administration. Transplant Direct. (2021) 7:e656. doi: 10.1097/TXD.0000000000001113 33490381 PMC7817285

[48] David P Mittelstädt A Kouhestani D Anthuber A Kahlert C Sohn K. Current applications of liquid biopsy in gastrointestinal cancer disease-from early cancer detection to individualized cancer treatment. Cancers (Basel). (2023) 15:1924. doi: 10.3390/cancers15071924 PMC10093361 37046585

[49] Roazzi L Patelli G Bencardino KB Amatu A Bonazzina E Tosi F. Ongoing clinical trials and future research scenarios of circulating tumor DNA for the treatment of metastatic colorectal cancer. Clin Colorectal Cancer. (2024) 23:295–308. doi: 10.1016/j.clcc.2024.02.001 38519391

[50] Lee MS Kaseb AO Pant S. The emerging role of circulating tumor DNA in non-colorectal gastrointestinal cancers. Clin Cancer Res. (2023) 29:3267–74. doi: 10.1158/1078-0432.CCR-22-3626 37092904

[51] Thompson JC Carpenter EL Silva BA Rosenstein J Chien AL Quinn K. Serial monitoring of circulating tumor DNA by next-generation gene sequencing as a biomarker of response and survival in patients with advanced NSCLC receiving pembrolizumab-based therapy. JCO Precis Oncol. (2021) 5:PO.20.00321. doi: 10.1200/PO.20.00321 PMC8169078 34095713

[52] Battaglin F Lenz HJ. Clinical applications of circulating tumor DNA profiling in GI cancers. JCO Oncol Pract. (2024) 20:1481–90. doi: 10.1200/OP.24.00167 PMC11567053 39531845

[53] Alexandrov LB Kim J Haradhvala NJ Huang MN Tian Ng AW Wu Y. The repertoire of mutational signatures in human cancer. Nature. (2020) 578:94–101. doi: 10.1038/s41586-020-1943-3 32025018 PMC7054213

[54] Tsai A Deshmukh SK Wu S Hoon D El-Deiry W Grothey A. (2025). Potential impact of non-exonuclease POLE and POLD1 mutations on high and ultrahigh TMB across cancers, in: ASCO Annual Meeting; Abstract number: e14558, Chicago, Illinois, USA, 6/1/2025.

