# Supplementary material for: Case Report: Metastatic small bowel adenocarcinoma with DNA mismatch repair deficiency in an organ transplant recipient treated with anti-PD-1 immunotherapy
Source: Front Oncol. 2025 Jun 12;15:1579364. doi: 10.3389/fonc.2025.1579364 (PMC12197958; doi:10.3389/fonc.2025.1579364)
Supplement: Supplementary file 1 [file Table1.docx]

**Supplement Table 1. Summary of Detected Somatic Alterations**

This table lists the somatic alterations detected from the patient’s initial Guardant 360 (83 gene panel) test.

| **Detected Alteration / Biomarker** | **Variant Allele Fraction (% cfDNA)** |
| --- | --- |
| *FGFR2* D101Y | 21.9 |
| *FGFR2* R759* | 21.7 |
| *JAK3* E481* | 21.5 |
| *ROS1* E1612* | 21.3 |
| *PDGFRA* E263* | 20.9 |
| *SMO* Splice Site SNV | 20.4 |
| *SMAD4* E520* | 20.2 |
| *KIT* Splice Site SNV | 20.1 |
| *BRCA2* E1110* | 20.0 |
| *JAK2* R235I | 20.0 |
| *MSH6* E1234* | 19.4 |
| *ATM* S571Y | 19.4 |
| *DDR2* K616N | 19.2 |
| *TERT* C54* | 19.1 |
| *CDH1* D786N | 19.0 |
| *ESR1* D426N | 18.7 |
| *RHEB* Splice Site SNV | 18.7 |
| *ROS1* E1244* | 18.5 |
| *DDR2* R611Q | 18.2 |
| *RB1* E746* | 18 |
| *JAK3* S568* | 17.6 |
| *MSH2* E580* | 17.5 |
| *CTNNB1* R587Q | 17.5 |
| *SMAD4* E53* | 17.4 |
| *KIT* E329* | 17.1 |
| *ROS1* Splice Site SNV | 16.4 |
| *FGFR3* E198K | 14.2 |
| *RB1* E137* | 13.9 |
| *STK11* D194N | 11.6 |
| *ESR1* S450Y | 7.0 |
| *PIK3CA* T1025A | 4.4 |
| *BRAF* D594E | 2.8 |
| *TP53* I232T | 2.7 |
| *FGFR2* L550I | 1.0 |
| *EGFR* N73K | 1.0 |
| *APC* R24* | 1.0 |
| *AR* A766T | 0.9 |
| *NF1* E524* | 0.7 |
| *BRCA1* S184A | 0.6 |
| *APC* S959Y | 0.6 |
| *NF1* E1264* | 0.5 |
| *KRAS* K117N | 0.5 |
| *GNAS* Q227H | 0.5 |
| *NF1* R1362* | 0.5 |
| *CDK12* Splice Site SNV | 0.5 |
| *BRCA2* E2129* | 0.4 |
| *BRAF* N580K | 0.4 |
| *ATM* R2598* | 0.4 |
| *JAK2* R588I | 0.4 |
| *ALK* S895Y | 0.4 |
| *APC* E129* | 0.3 |
| *ATM* E277* | 0.3 |
| *PDGFRA* E301* | 0.3 |
| *BRAF* N581T | 0.3 |
| *TP53* R213* | 0.3 |
| *NF1* S1489G | 0.3 |
| *FANCA* S83Y | 0.3 |
| *NF1* Splice Site SNV | 0.3 |
| *IDH2* D390Y | 0.2 |
| *JAK2* E1080* | 0.2 |
| *NF1* E1220* | 0.2 |
| *MSH6* E1322* | 0.2 |
| *RB1* E237* | 0.2 |
| *BRCA2* E97* | 0.2 |
| *JAK2* F1061C | 0.2 |
| *KRAS* L19F | 0.2 |
| *JAK2* L712I | 0.2 |
| *FGFR2* L761I | 0.2 |
| *NF1* N78fs* | 0.2 |
| *ALK* P837S | 0.2 |
| *PTPN11* Q510H | 0.2 |
| *APC* R2237* | 0.2 |
| *FANCA* R272I | 0.2 |
| *NF1* R440* | 0.2 |
| *FBXW7* R689W | 0.2 |
| *BRCA1* E1112* | 0.1 |
| *ROS1* E2201* | 0.1 |
| *PTPN11* E400* | 0.1 |
| *NF1* E595* | 0.1 |
| *BRCA1* E720* | 0.1 |
| *ALK* H384Q | 0.1 |
| *IDH2* K280N | 0.1 |
| *MAP2K2* L102R | 0.1 |
| *EGFR* L140V | 0.1 |
| *EGFR* L450* | 0.1 |
| *APC* N1797fs* | 0.1 |
| *FGFR2* N549H | 0.1 |
| *GNAQ* R183* | 0.1 |
| *ARID1A* R1989* | 0.1 |
| *BRCA2* R2520Q | 0.1 |
| *BRCA2* S3319Y | 0.1 |
| *ALK* Splice Site SNV | 0.1 |
| *NF1* Splice Site SNV | 0.1 |
| *ROS1* Splice Site SNV | 0.1 |
| *ERBB2* D769Y | 0.09 |
| *APC* R1114* | 0.09 |
| *TP53* R267W | 0.09 |
| *GNAS* R201H | 0.06 |
| *PIK3CA* R88Q | 0.06 |
| *PTPN11* D61Y | 0.05 |
| *PIK3CA* M1043I | 0.05 |

*Nonsense mutation: A point mutation that results in a premature stop at that codon.

fs* Frameshift mutation: A mutation that results in a shift of the open reading frame followed by a premature stop codon.
